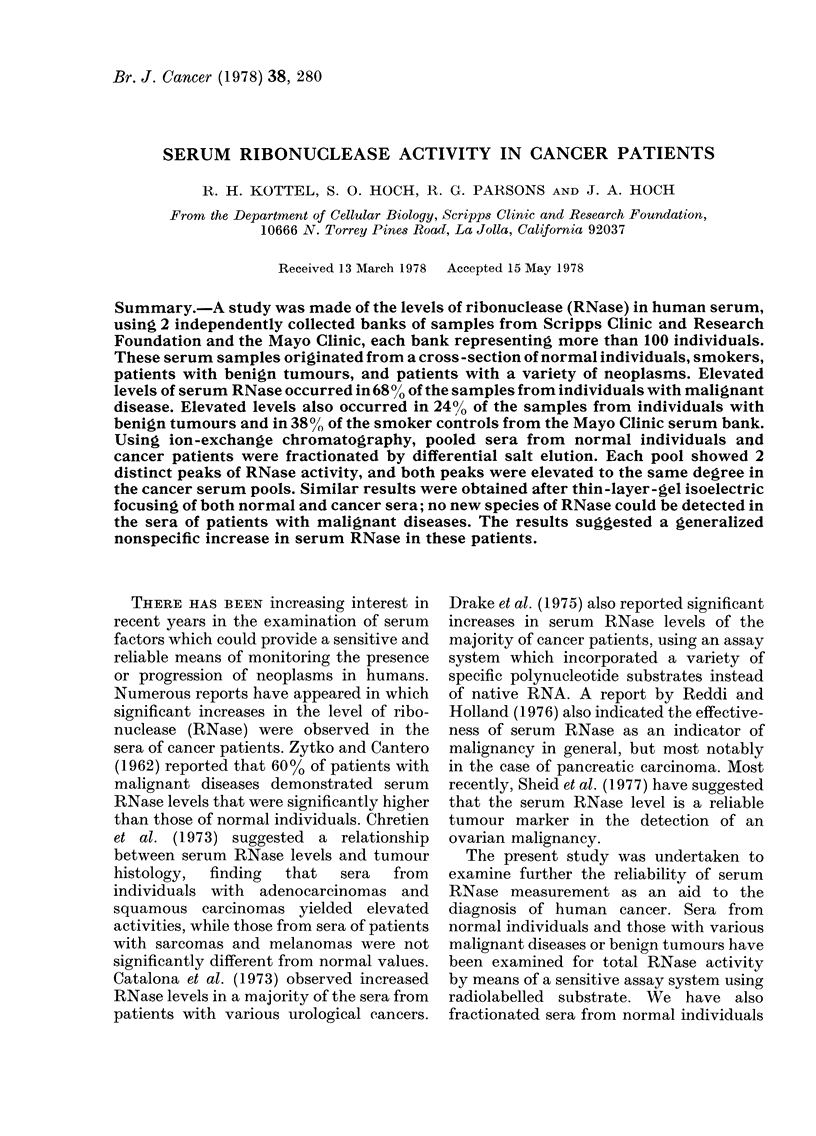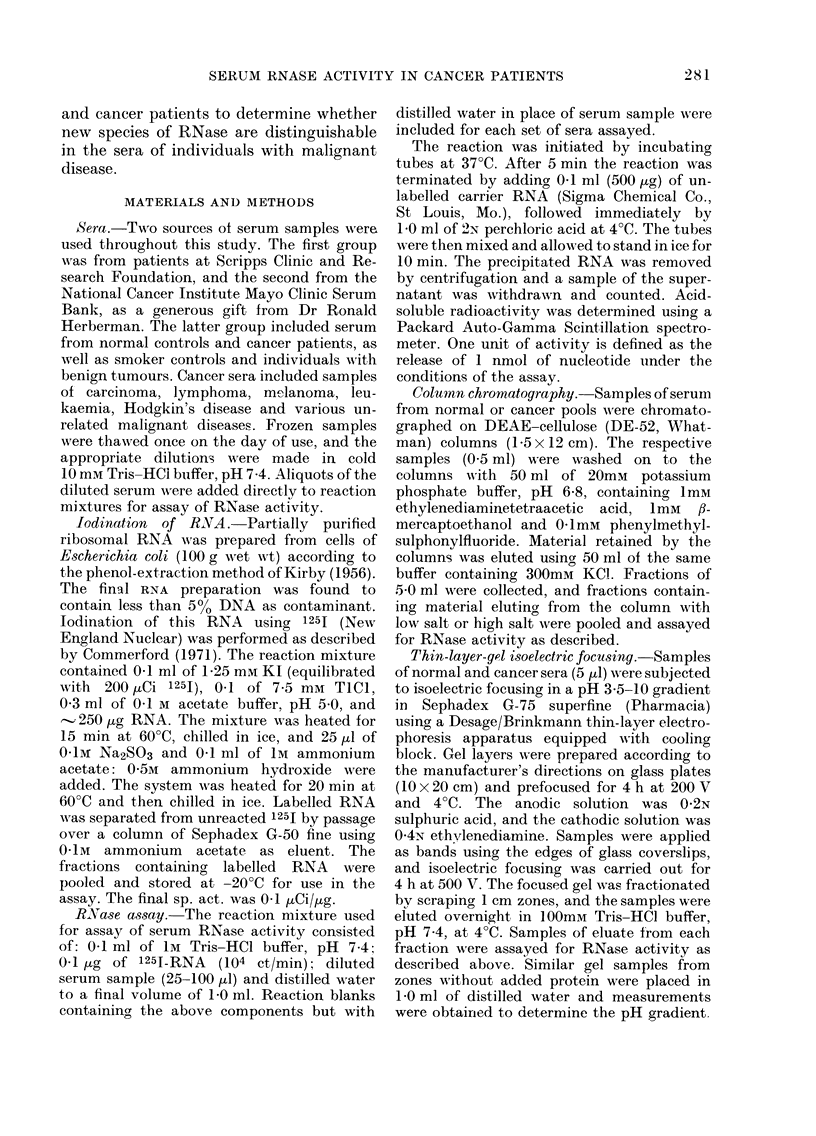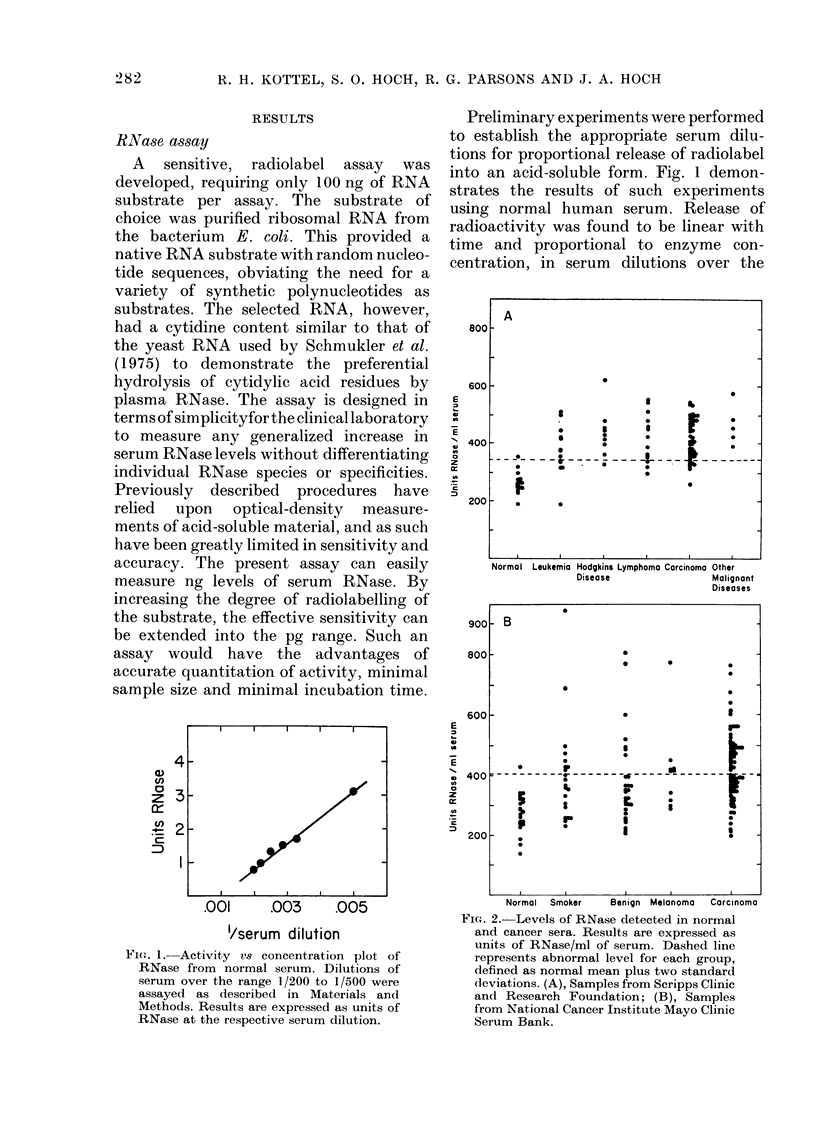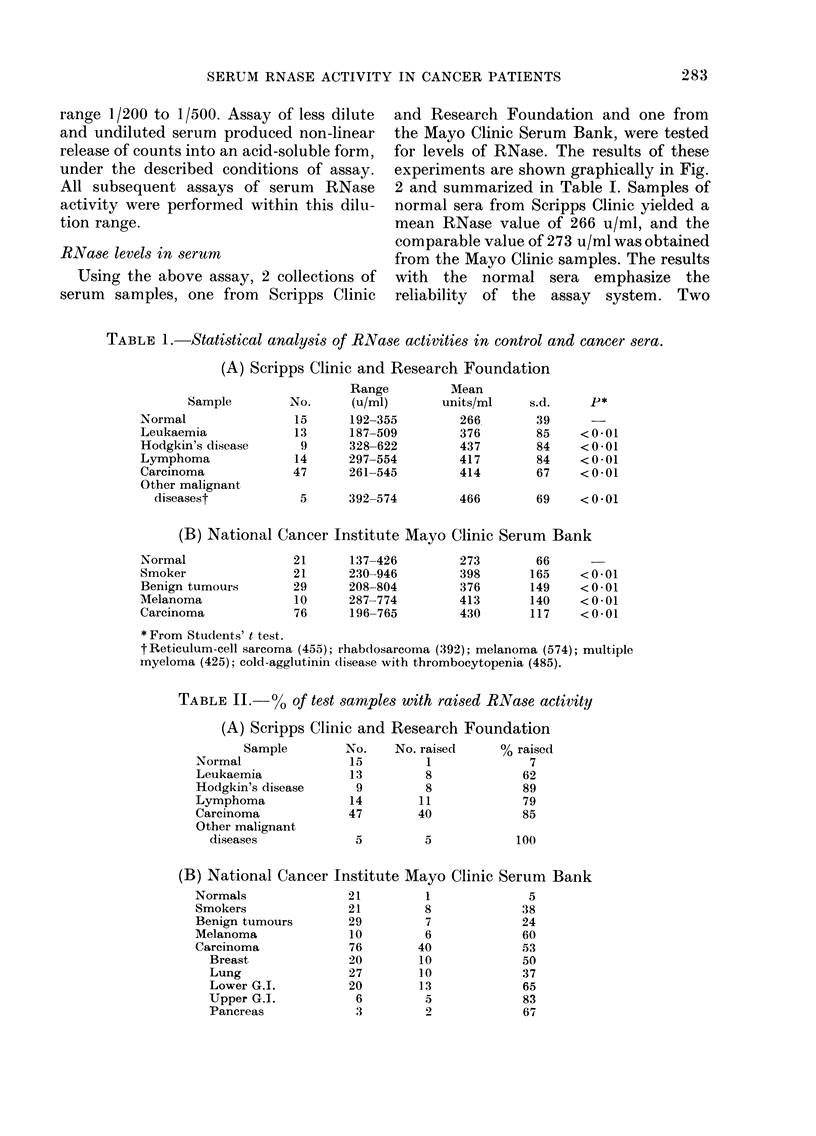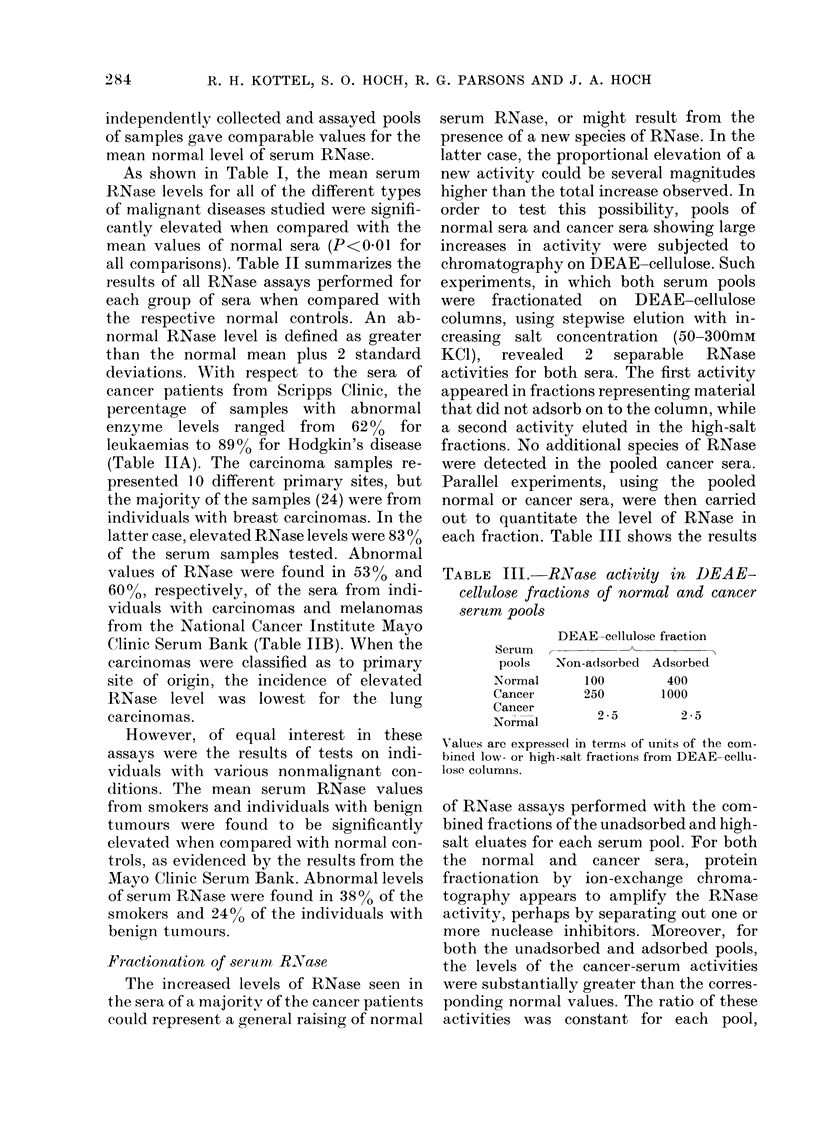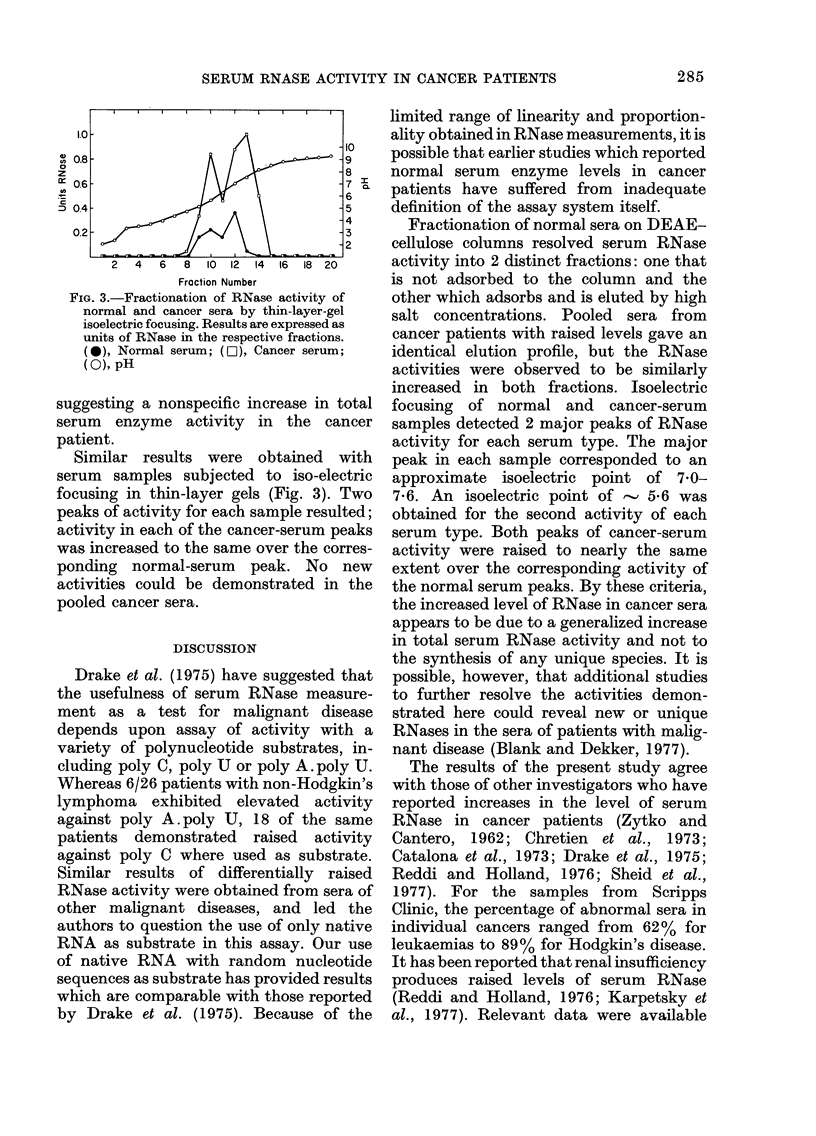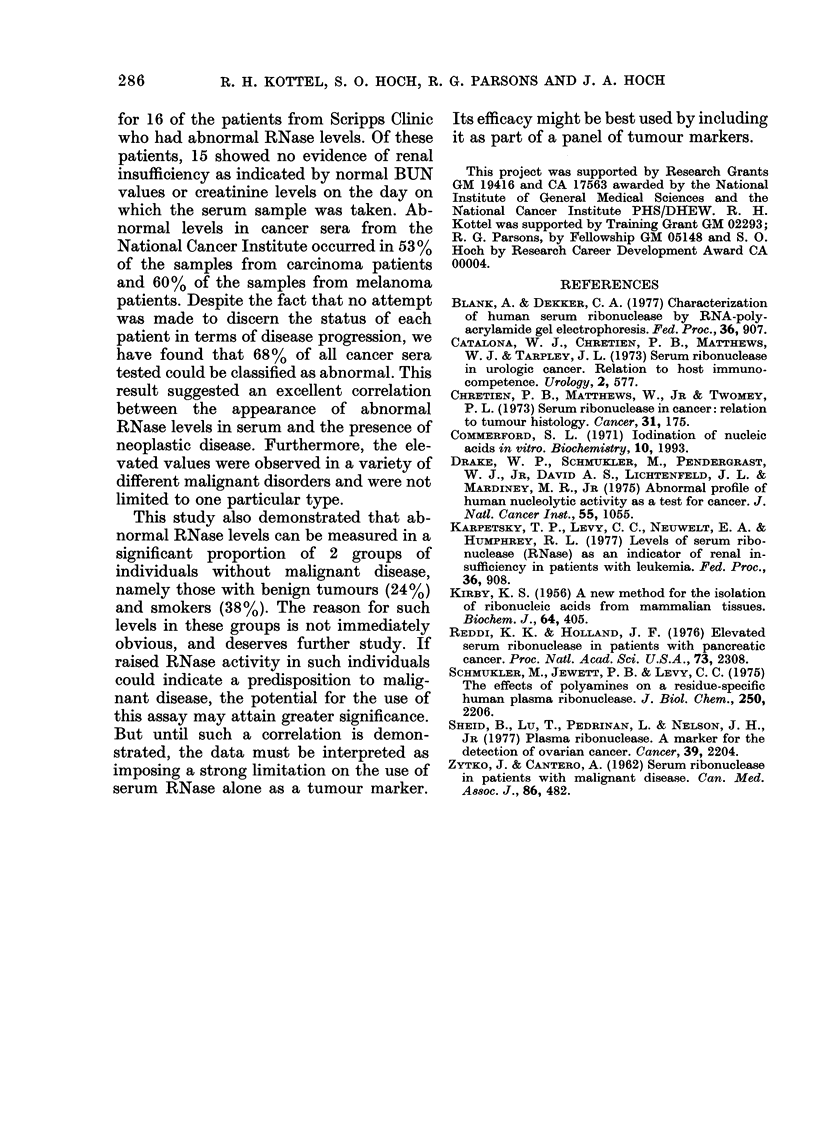# Serum ribonuclease activity in cancer patients.

**DOI:** 10.1038/bjc.1978.199

**Published:** 1978-08

**Authors:** R. H. Kottel, S. O. Hoch, R. G. Parsons, J. A. Hoch

## Abstract

A study was made of the levels of ribonuclease (RNase) in human serum, using 2 independently collected banks of samples from Scripps Clinic and Research Foundation and the Mayo Clinic, each bank representing more than 100 individuals. These serum samples originated from a cross-section of normal individuals, smokers, patients with benign tumours, and patients with a variety of neoplasms. Elevated levels of serum RNase occurred in 68% of the samples from individuals with malignant disease. Elevated levels also occurred in 24% of the samples from individuals with benign tumours and in 38% of the smoker controls from the Mayo Clinic serum bank. Using ion-exchange chromatography, pooled sera from normal individuals and cancer patients were fractionated by differential salt elution. Each pool showed 2 distinct peaks of RNase activity, and both peaks were elevated to the same degree in the cancer serum pools. Similar results were obtained after thin-layer-gel isoelectric focusing of both normal and cancer sera; no new species of RNase could be detected in the sera of patients with malignant diseases. The results suggested a generalized nonspecific increase in serum RNase in these patients.


*Br. J. Cancer* (1978) **38**, 280

### SERUM RIBONUCLEASE ACTIVITY IN CANCER PATIENTS

R. H. KOTTEL, S. O. HOCH, R. G. PARSONS AND J. A. HOCH

*From the Department of Cellular Biology, Scripps Clinic and Research Foundation,*
10666 *N. Torrey Pines Road, La Jolla, California* 92037

Received 13 March 1978 Accepted 15 May 1978

**Summary.—A study was made of the levels of ribonuclease (RNase) in human serum, using 2 independently collected banks of samples from Scripps Clinic and Research Foundation and the Mayo Clinic, each bank representing more than 100 individuals. These serum samples originated from a cross-section of normal individuals, smokers, patients with benign tumours, and patients with a variety of neoplasms. Elevated levels of serum RNase occurred in 68% of the samples from individuals with malignant disease. Elevated levels also occurred in 24% of the samples from individuals with benign tumours and in 38% of the smoker controls from the Mayo Clinic serum bank. Using ion-exchange chromatography, pooled sera from normal individuals and cancer patients were fractionated by differential salt elution. Each pool showed 2 distinct peaks of RNase activity, and both peaks were elevated to the same degree in the cancer serum pools. Similar results were obtained after thin-layer-gel isoelectric focusing of both normal and cancer sera; no new species of RNase could be detected in the sera of patients with malignant diseases. The results suggested a generalized nonspecific increase in serum RNase in these patients.**

THERE HAS BEEN increasing interest in recent years in the examination of serum factors which could provide a sensitive and reliable means of monitoring the presence or progression of neoplasms in humans. Numerous reports have appeared in which significant increases in the level of ribonuclease (RNase) were observed in the sera of cancer patients. Zytko and Cantero (1962) reported that 60% of patients with malignant diseases demonstrated serum RNase levels that were significantly higher than those of normal individuals. Chretien *et al.* (1973) suggested a relationship between serum RNase levels and tumour histology, finding that sera from individuals with adenocarcinomas and squamous carcinomas yielded elevated activities, while those from sera of patients with sarcomas and melanomas were not significantly different from normal values. Catalona *et al.* (1973) observed increased RNase levels in a majority of the sera from patients with various urological cancers. Drake *et al.* (1975) also reported significant increases in serum RNase levels of the majority of cancer patients, using an assay system which incorporated a variety of specific polynucleotide substrates instead of native RNA. A report by Reddi and Holland (1976) also indicated the effectiveness of serum RNase as an indicator of malignancy in general, but most notably in the case of pancreatic carcinoma. Most recently, Sheid *et al.* (1977) have suggested that the serum RNase level is a reliable tumour marker in the detection of an ovarian malignancy.

The present study was undertaken to examine further the reliability of serum RNase measurement as an aid to the diagnosis of human cancer. Sera from normal individuals and those with various malignant diseases or benign tumours have been examined for total RNase activity by means of a sensitive assay system using radiolabelled substrate. We have also fractionated sera from normal individuals and cancer patients to determine whether new species of RNase are distinguishable in the sera of individuals with malignant disease.

### MATERIALS AND METHODS

*Sera.*—Two sources of serum samples were used throughout this study. The first group was from patients at Scripps Clinic and Research Foundation, and the second from the National Cancer Institute Mayo Clinic Serum Bank, as a generous gift from Dr Ronald Herberman. The latter group included serum from normal controls and cancer patients, as well as smoker controls and individuals with benign tumours. Cancer sera included samples of carcinoma, lymphoma, melanoma, leukaemia, Hodgkin's disease and various unrelated malignant diseases. Frozen samples were thawed once on the day of use, and the appropriate dilutions were made in cold 10 mM Tris–HCl buffer, pH 7·4. Aliquots of the diluted serum were added directly to reaction mixtures for assay of RNase activity.

*Iodination of RNA.*—Partially purified ribosomal RNA was prepared from cells of *Escherichia coli* (100 g wet wt) according to the phenol-extraction method of Kirby (1956). The final RNA preparation was found to contain less than 5% DNA as contaminant. Iodination of this RNA using $^{125}I$ (New England Nuclear) was performed as described by Commerford (1971). The reaction mixture contained 0·1 ml of 1·25 mM KI (equilibrated with 200 $\mu$Ci $^{125}I$), 0·1 of 7·5 mM TlCl, 0·3 ml of 0·1 M acetate buffer, pH 5·0, and $\sim$250 $\mu$g RNA. The mixture was heated for 15 min at 60°C, chilled in ice, and 25 $\mu$l of 0·1M $Na_2SO_3$ and 0·1 ml of 1M ammonium acetate: 0·5M ammonium hydroxide were added. The system was heated for 20 min at 60°C and then chilled in ice. Labelled RNA was separated from unreacted $^{125}I$ by passage over a column of Sephadex G-50 fine using 0·1M ammonium acetate as eluent. The fractions containing labelled RNA were pooled and stored at −20°C for use in the assay. The final sp. act. was 0·1 $\mu$Ci/$\mu$g.

*RNase assay.*—The reaction mixture used for assay of serum RNase activity consisted of: 0·1 ml of 1M Tris–HCl buffer, pH 7·4; 0·1 $\mu$g of $^{125}I$-RNA ($10^4$ ct/min); diluted serum sample (25–100 $\mu$l) and distilled water to a final volume of 1·0 ml. Reaction blanks containing the above components but with distilled water in place of serum sample were included for each set of sera assayed.

The reaction was initiated by incubating tubes at 37°C. After 5 min the reaction was terminated by adding 0·1 ml (500 $\mu$g) of unlabelled carrier RNA (Sigma Chemical Co., St Louis, Mo.), followed immediately by 1·0 ml of 2N perchloric acid at 4°C. The tubes were then mixed and allowed to stand in ice for 10 min. The precipitated RNA was removed by centrifugation and a sample of the supernatant was withdrawn and counted. Acid-soluble radioactivity was determined using a Packard Auto-Gamma Scintillation spectrometer. One unit of activity is defined as the release of 1 nmol of nucleotide under the conditions of the assay.

*Column chromatography.*—Samples of serum from normal or cancer pools were chromatographed on DEAE–cellulose (DE-52, Whatman) columns (1·5 × 12 cm). The respective samples (0·5 ml) were washed on to the columns with 50 ml of 20mM potassium phosphate buffer, pH 6·8, containing 1mM ethylenediaminetetraacetic acid, 1mM $\beta$-mercaptoethanol and 0·1mM phenylmethylsulphonylfluoride. Material retained by the columns was eluted using 50 ml of the same buffer containing 300mM KCl. Fractions of 5·0 ml were collected, and fractions containing material eluting from the column with low salt or high salt were pooled and assayed for RNase activity as described.

*Thin-layer-gel isoelectric focusing.*—Samples of normal and cancer sera (5 $\mu$l) were subjected to isoelectric focusing in a pH 3·5–10 gradient in Sephadex G-75 superfine (Pharmacia) using a Desage/Brinkmann thin-layer electrophoresis apparatus equipped with cooling block. Gel layers were prepared according to the manufacturer's directions on glass plates (10 × 20 cm) and prefocused for 4 h at 200 V and 4°C. The anodic solution was 0·2N sulphuric acid, and the cathodic solution was 0·4N ethylenediamine. Samples were applied as bands using the edges of glass coverslips, and isoelectric focusing was carried out for 4 h at 500 V. The focused gel was fractionated by scraping 1 cm zones, and the samples were eluted overnight in 100mM Tris–HCl buffer, pH 7·4, at 4°C. Samples of eluate from each fraction were assayed for RNase activity as described above. Similar gel samples from zones without added protein were placed in 1·0 ml of distilled water and measurements were obtained to determine the pH gradient.

### RESULTS

### *RNase assay*

A sensitive, radiolabel assay was developed, requiring only 100 ng of RNA substrate per assay. The substrate of choice was purified ribosomal RNA from the bacterium *E. coli.* This provided a native RNA substrate with random nucleotide sequences, obviating the need for a variety of synthetic polynucleotides as substrates. The selected RNA, however, had a cytidine content similar to that of the yeast RNA used by Schmukler *et al.* (1975) to demonstrate the preferential hydrolysis of cytidylic acid residues by plasma RNase. The assay is designed in terms of simplicity for the clinical laboratory to measure any generalized increase in serum RNase levels without differentiating individual RNase species or specificities. Previously described procedures have relied upon optical-density measurements of acid-soluble material, and as such have been greatly limited in sensitivity and accuracy. The present assay can easily measure ng levels of serum RNase. By increasing the degree of radiolabelling of the substrate, the effective sensitivity can be extended into the pg range. Such an assay would have the advantages of accurate quantitation of activity, minimal sample size and minimal incubation time.

FIG. 1.—Activity *vs* concentration plot of RNase from normal serum. Dilutions of serum over the range 1/200 to 1/500 were assayed as described in Materials and Methods. Results are expressed as units of RNase at the respective serum dilution.

Preliminary experiments were performed to establish the appropriate serum dilutions for proportional release of radiolabel into an acid-soluble form. Fig. 1 demonstrates the results of such experiments using normal human serum. Release of radioactivity was found to be linear with time and proportional to enzyme concentration, in serum dilutions over the

FIG. 2.—Levels of RNase detected in normal and cancer sera. Results are expressed as units of RNase/ml of serum. Dashed line represents abnormal level for each group, defined as normal mean plus two standard deviations. (A), Samples from Scripps Clinic and Research Foundation; (B), Samples from National Cancer Institute Mayo Clinic Serum Bank.

range 1/200 to 1/500. Assay of less dilute and undiluted serum produced non-linear release of counts into an acid-soluble form, under the described conditions of assay. All subsequent assays of serum RNase activity were performed within this dilution range.

*RNase levels in serum*

Using the above assay, 2 collections of serum samples, one from Scripps Clinic and Research Foundation and one from the Mayo Clinic Serum Bank, were tested for levels of RNase. The results of these experiments are shown graphically in Fig. 2 and summarized in Table I. Samples of normal sera from Scripps Clinic yielded a mean RNase value of 266 u/ml, and the comparable value of 273 u/ml was obtained from the Mayo Clinic samples. The results with the normal sera emphasize the reliability of the assay system. Two

TABLE 1.—*Statistical analysis of RNase activities in control and cancer sera.*

(A) Scripps Clinic and Research Foundation

| Sample | No. | Range (u/ml) | Mean units/ml | s.d. | $P$* |
|---|---|---|---|---|---|
| Normal | 15 | 192–355 | 266 | 39 | — |
| Leukaemia | 13 | 187–509 | 376 | 85 | <0·01 |
| Hodgkin's disease | 9 | 328–622 | 437 | 84 | <0·01 |
| Lymphoma | 14 | 297–554 | 417 | 84 | <0·01 |
| Carcinoma | 47 | 261–545 | 414 | 67 | <0·01 |
| Other malignant diseases† | 5 | 392–574 | 466 | 69 | <0·01 |

(B) National Cancer Institute Mayo Clinic Serum Bank

| Sample | No. | Range (u/ml) | Mean units/ml | s.d. | $P$* |
|---|---|---|---|---|---|
| Normal | 21 | 137–426 | 273 | 66 | — |
| Smoker | 21 | 230–946 | 398 | 165 | <0·01 |
| Benign tumours | 29 | 208–804 | 376 | 149 | <0·01 |
| Melanoma | 10 | 287–774 | 413 | 140 | <0·01 |
| Carcinoma | 76 | 196–765 | 430 | 117 | <0·01 |

* From Students' $t$ test.

† Reticulum-cell sarcoma (455); rhabdosarcoma (392); melanoma (574); multiple myeloma (425); cold-agglutinin disease with thrombocytopenia (485).

TABLE II.—*% of test samples with raised RNase activity*

(A) Scripps Clinic and Research Foundation

| Sample | No. | No. raised | % raised |
|---|---|---|---|
| Normal | 15 | 1 | 7 |
| Leukaemia | 13 | 8 | 62 |
| Hodgkin's disease | 9 | 8 | 89 |
| Lymphoma | 14 | 11 | 79 |
| Carcinoma | 47 | 40 | 85 |
| Other malignant diseases | 5 | 5 | 100 |

(B) National Cancer Institute Mayo Clinic Serum Bank

| Sample | No. | No. raised | % raised |
|---|---|---|---|
| Normals | 21 | 1 | 5 |
| Smokers | 21 | 8 | 38 |
| Benign tumours | 29 | 7 | 24 |
| Melanoma | 10 | 6 | 60 |
| Carcinoma | 76 | 40 | 53 |
| Breast | 20 | 10 | 50 |
| Lung | 27 | 10 | 37 |
| Lower G.I. | 20 | 13 | 65 |
| Upper G.I. | 6 | 5 | 83 |
| Pancreas | 3 | 2 | 67 |

independently collected and assayed pools of samples gave comparable values for the mean normal level of serum RNase.

As shown in Table I, the mean serum RNase levels for all of the different types of malignant diseases studied were significantly elevated when compared with the mean values of normal sera ($P<0{\cdot}01$ for all comparisons). Table II summarizes the results of all RNase assays performed for each group of sera when compared with the respective normal controls. An abnormal RNase level is defined as greater than the normal mean plus 2 standard deviations. With respect to the sera of cancer patients from Scripps Clinic, the percentage of samples with abnormal enzyme levels ranged from 62% for leukaemias to 89% for Hodgkin's disease (Table IIA). The carcinoma samples represented 10 different primary sites, but the majority of the samples (24) were from individuals with breast carcinomas. In the latter case, elevated RNase levels were 83% of the serum samples tested. Abnormal values of RNase were found in 53% and 60%, respectively, of the sera from individuals with carcinomas and melanomas from the National Cancer Institute Mayo Clinic Serum Bank (Table IIB). When the carcinomas were classified as to primary site of origin, the incidence of elevated RNase level was lowest for the lung carcinomas.

However, of equal interest in these assays were the results of tests on individuals with various nonmalignant conditions. The mean serum RNase values from smokers and individuals with benign tumours were found to be significantly elevated when compared with normal controls, as evidenced by the results from the Mayo Clinic Serum Bank. Abnormal levels of serum RNase were found in 38% of the smokers and 24% of the individuals with benign tumours.

*Fractionation of serum RNase*

The increased levels of RNase seen in the sera of a majority of the cancer patients could represent a general raising of normal serum RNase, or might result from the presence of a new species of RNase. In the latter case, the proportional elevation of a new activity could be several magnitudes higher than the total increase observed. In order to test this possibility, pools of normal sera and cancer sera showing large increases in activity were subjected to chromatography on DEAE–cellulose. Such experiments, in which both serum pools were fractionated on DEAE–cellulose columns, using stepwise elution with increasing salt concentration (50–300mM KCl), revealed 2 separable RNase activities for both sera. The first activity appeared in fractions representing material that did not adsorb on to the column, while a second activity eluted in the high-salt fractions. No additional species of RNase were detected in the pooled cancer sera. Parallel experiments, using the pooled normal or cancer sera, were then carried out to quantitate the level of RNase in each fraction. Table III shows the results

TABLE III.—*RNase activity in DEAE–cellulose fractions of normal and cancer serum pools*

| Serum pools | DEAE–cellulose fraction | |
|---|---|---|
| | Non-adsorbed | Adsorbed |
| Normal | 100 | 400 |
| Cancer | 250 | 1000 |
| $\frac{\text{Cancer}}{\text{Normal}}$ | 2·5 | 2·5 |

Values are expressed in terms of units of the combined low- or high-salt fractions from DEAE–cellulose columns.

of RNase assays performed with the combined fractions of the unadsorbed and high-salt eluates for each serum pool. For both the normal and cancer sera, protein fractionation by ion-exchange chromatography appears to amplify the RNase activity, perhaps by separating out one or more nuclease inhibitors. Moreover, for both the unadsorbed and adsorbed pools, the levels of the cancer-serum activities were substantially greater than the corresponding normal values. The ratio of these activities was constant for each pool,

FIG. 3.—Fractionation of RNase activity of normal and cancer sera by thin-layer-gel isoelectric focusing. Results are expressed as units of RNase in the respective fractions. (●), Normal serum; (□), Cancer serum; (○), pH

suggesting a nonspecific increase in total serum enzyme activity in the cancer patient.

Similar results were obtained with serum samples subjected to iso-electric focusing in thin-layer gels (Fig. 3). Two peaks of activity for each sample resulted; activity in each of the cancer-serum peaks was increased to the same over the corresponding normal-serum peak. No new activities could be demonstrated in the pooled cancer sera.

### DISCUSSION

Drake *et al.* (1975) have suggested that the usefulness of serum RNase measurement as a test for malignant disease depends upon assay of activity with a variety of polynucleotide substrates, including poly C, poly U or poly A.poly U. Whereas 6/26 patients with non-Hodgkin's lymphoma exhibited elevated activity against poly A.poly U, 18 of the same patients demonstrated raised activity against poly C where used as substrate. Similar results of differentially raised RNase activity were obtained from sera of other malignant diseases, and led the authors to question the use of only native RNA as substrate in this assay. Our use of native RNA with random nucleotide sequences as substrate has provided results which are comparable with those reported by Drake *et al.* (1975). Because of the limited range of linearity and proportionality obtained in RNase measurements, it is possible that earlier studies which reported normal serum enzyme levels in cancer patients have suffered from inadequate definition of the assay system itself.

Fractionation of normal sera on DEAE–cellulose columns resolved serum RNase activity into 2 distinct fractions: one that is not adsorbed to the column and the other which adsorbs and is eluted by high salt concentrations. Pooled sera from cancer patients with raised levels gave an identical elution profile, but the RNase activities were observed to be similarly increased in both fractions. Isoelectric focusing of normal and cancer-serum samples detected 2 major peaks of RNase activity for each serum type. The major peak in each sample corresponded to an approximate isoelectric point of 7·0–7·6. An isoelectric point of ~ 5·6 was obtained for the second activity of each serum type. Both peaks of cancer-serum activity were raised to nearly the same extent over the corresponding activity of the normal serum peaks. By these criteria, the increased level of RNase in cancer sera appears to be due to a generalized increase in total serum RNase activity and not to the synthesis of any unique species. It is possible, however, that additional studies to further resolve the activities demonstrated here could reveal new or unique RNases in the sera of patients with malignant disease (Blank and Dekker, 1977).

The results of the present study agree with those of other investigators who have reported increases in the level of serum RNase in cancer patients (Zytko and Cantero, 1962; Chretien *et al.*, 1973; Catalona *et al.*, 1973; Drake *et al.*, 1975; Reddi and Holland, 1976; Sheid *et al.*, 1977). For the samples from Scripps Clinic, the percentage of abnormal sera in individual cancers ranged from 62% for leukaemias to 89% for Hodgkin's disease. It has been reported that renal insufficiency produces raised levels of serum RNase (Reddi and Holland, 1976; Karpetsky *et al.*, 1977). Relevant data were available for 16 of the patients from Scripps Clinic who had abnormal RNase levels. Of these patients, 15 showed no evidence of renal insufficiency as indicated by normal BUN values or creatinine levels on the day on which the serum sample was taken. Abnormal levels in cancer sera from the National Cancer Institute occurred in 53% of the samples from carcinoma patients and 60% of the samples from melanoma patients. Despite the fact that no attempt was made to discern the status of each patient in terms of disease progression, we have found that 68% of all cancer sera tested could be classified as abnormal. This result suggested an excellent correlation between the appearance of abnormal RNase levels in serum and the presence of neoplastic disease. Furthermore, the elevated values were observed in a variety of different malignant disorders and were not limited to one particular type.

This study also demonstrated that abnormal RNase levels can be measured in a significant proportion of 2 groups of individuals without malignant disease, namely those with benign tumours (24%) and smokers (38%). The reason for such levels in these groups is not immediately obvious, and deserves further study. If raised RNase activity in such individuals could indicate a predisposition to malignant disease, the potential for the use of this assay may attain greater significance. But until such a correlation is demonstrated, the data must be interpreted as imposing a strong limitation on the use of serum RNase alone as a tumour marker. Its efficacy might be best used by including it as part of a panel of tumour markers.

This project was supported by Research Grants GM 19416 and CA 17563 awarded by the National Institute of General Medical Sciences and the National Cancer Institute PHS/DHEW. R. H. Kottel was supported by Training Grant GM 02293; R. G. Parsons, by Fellowship GM 05148 and S. O. Hoch by Research Career Development Award CA 00004.

### REFERENCES

Blank, A. & Dekker, C. A. (1977) Characterization of human serum ribonuclease by RNA-polyacrylamide gel electrophoresis. *Fed. Proc.*, **36**, 907.

Catalona, W. J., Chretien, P. B., Matthews, W. J. & Tarpley, J. L. (1973) Serum ribonuclease in urologic cancer. Relation to host immunocompetence. *Urology*, **2**, 577.

Chretien, P. B., Matthews, W., Jr & Twomey, P. L. (1973) Serum ribonuclease in cancer: relation to tumour histology. *Cancer*, **31**, 175.

Commerford, S. L. (1971) Iodination of nucleic acids *in vitro*. *Biochemistry*, **10**, 1993.

Drake, W. P., Schmukler, M., Pendergrast, W. J., Jr, David A. S., Lichtenfeld, J. L. & Mardiney, M. R., Jr (1975) Abnormal profile of human nucleolytic activity as a test for cancer. *J. Natl. Cancer Inst.*, **55**, 1055.

Karpetsky, T. P., Levy, C. C., Neuwelt, E. A. & Humphrey, R. L. (1977) Levels of serum ribonuclease (RNase) as an indicator of renal insufficiency in patients with leukemia. *Fed. Proc.*, **36**, 908.

Kirby, K. S. (1956) A new method for the isolation of ribonucleic acids from mammalian tissues. *Biochem. J.*, **64**, 405.

Reddi, K. K. & Holland, J. F. (1976) Elevated serum ribonuclease in patients with pancreatic cancer. *Proc. Natl. Acad. Sci. U.S.A.*, **73**, 2308.

Schmukler, M., Jewett, P. B. & Levy, C. C. (1975) The effects of polyamines on a residue-specific human plasma ribonuclease. *J. Biol. Chem.*, **250**, 2206.

Sheid, B., Lu, T., Pedrinan, L. & Nelson, J. H., Jr (1977) Plasma ribonuclease. A marker for the detection of ovarian cancer. *Cancer*, **39**, 2204.

Zytko, J. & Cantero, A. (1962) Serum ribonuclease in patients with malignant disease. *Can. Med. Assoc. J.*, **86**, 482.